# Encysted Tumor of Neck

**Published:** 1874-01

**Authors:** C. W. McMillen


Encysted Tumor of the Neck—*By C. W. McMillen, M. D.*—A well-matured male child, twenty-two months old. The tumor occupied the left side of the neck, extending from the median line anteriorly to within one inch of the spine posteriorly, forcing the head entirely over on the right shoulder. I introduced a narrow straight bistoury, making an opening sufficiently large to permit all the fluid to escape. I explored the posterior sac with the grooved needle, and was surprised to find a thick grumous coffee-ground fluid. I punctured this sac also, with the bistoury, completely emptying it of its contents. This treatment gave the child great relief for two or three months, when the sac having refilled and increased in size, I determined to operate for a radical cure, it now being four months from the date of tapping. I introduced a double strand of surgeon's silk, by making a small incision two inches posterior to and through the partition, and out the same distance anteriorly; I passed the thread with an eyed probe. The fluid passed from the openings around the thread for a few days, when the tumor began to inflame, and drops of pus appeared at the openings. I continued the seton until satisfied that the internal surface of the sac was sufficiently inflamed, and suppuration fully set up. I punctured the sac, permitting the escape of a large amount of offensive pus, the walls collapsed, and the little patient was entirely relieved.

